# The Effects of Transcranial Direct Current Stimulation (tDCS) Combined With Proprioceptive Training for Blind Individuals: The Study Protocol for a Randomized Controlled Clinical Trial

**DOI:** 10.3389/fneur.2020.592376

**Published:** 2020-11-16

**Authors:** Rodolfo Borges Parreira, Jamile Benite Palma Lopes, Milena Santana França, Mayara Bernardo Albuquerque, Lorraine Barbosa Cordeiro, Deborah Carvalho da Silva Cardoso, Veronica Cimolin, Manuela Galli, Claudia Santos Oliveira

**Affiliations:** ^1^Master's and Doctoral Program in Health Sciences, Faculty of Medical Sciences, Santa Casa de São Paulo, São Paulo, Brazil; ^2^PostureLab, Paris, France; ^3^Department of Physical Therapy, University Center of Anápolis, Anápolis, Brazil; ^4^Master's and Doctoral Program in Human Movement and Rehabilitation, University Center of Anápolis, Anápolis, Brazil; ^5^Department of Electronics, Information and Bioengineering, Politecnico di Milano, Milan, Italy

**Keywords:** transcranial direct current stimulation, impaired vision, postural control, proprioceptive exercises, tDCS, rehabilitation

## Abstract

To maintain the balance, the postural system needs to integrate the three main sensorial systems: visual, vestibular, and somatosensory to keep postural control within the limits of stabilization. Damage of one of these systems, in this case, the vision, will have a great disturbance on the postural control influencing the behavior of the balance, resulting in falls. The aim of this study protocol for a randomized, controlled clinical trial is to analyze the effects of transcranial direct current stimulation (tDCS) combined with proprioceptive exercises on postural control in individuals with congenital and acquired blindness. In this randomized, controlled, double-blind, clinical trial, male, and female individuals with blindness between 18 and 55 years of age will participate in this study divided into three phases: 1—Determine differences in postural control and gait between individuals with congenital and acquired blindness with and without the use of a guide stick when wearing shoes and when barefoot; 2—A pilot study to analyze the effects a bilateral cerebellar anodal tDCS on postural on postural control and gait; and 3—A treatment protocol will be conducted in which the participants will be allocated to four groups: G1—active tDCS + dynamic proprioceptive exercises; G2—sham tDCS + dynamic proprioceptive exercises; G3—active tDCS + static proprioceptive exercises; and G4—sham tDCS + static proprioceptive exercises. Evaluations will involve a camera system for three-dimensional gait analysis, a force plate, and electromyography. Dynamic stability will be determined using the Timed Up and Go test and static stability will be analyzed with the aid of the force plate. The viability of this study will allow the determination of differences in postural control between individuals with congenital and acquired blindness, the analysis of the effect of tDCS on postural control, and the establishment of a rehabilitation protocol.

## Introduction

Postural control (PC) can be negatively affected in individuals with blindness due to the lack of availability of visual input (1, 2). The proprioceptive system cannot completely compensate this poor balance for a lack of vision even on high demand for physical activity such as sports participation (3, 4). Contributing factors in blind subjects include a lack of adequate information on the surrounding environment, spatial localization, and the orientation of body parts, which serve as a reference for perception and action in relation to the outside world (2).

Balance training or postural perturbation training, enhance proxies of static and dynamic steady-state, reactive balance, and dynamic reflex responses that induce disturbances in the sensory feedback system (5) to generate an automatic response in the neuromuscular control of a given joint. These interventions attempts to enhance PC and balance to improve the stability of the body, thereby reducing the risk of falls. A suggestion is that the central nervous system stimulates the dynamic behavior of the motor system for planning, control, and learning, integrating afferent and efferent sensory inputs during a proprioceptive activity in the absence of sight (6). Therefore, therapies that directly affect the higher centers of the central nervous system, in case of the motor cortical areas could lead to more effective results, and, transcranial direct current stimulation (tDCS) could be a way to achieve such neural changes directly.

Studies reported positive clinical effects with the use of tDCS combined with rehabilitation programs. In a meta-analysis (7) tDCS improved PC through the modulation of cortical excitability, which this technique could be a choice to enhance functionality and independence, and thus, reduce the rate of falls in individuals with a balance disorder, mainly on primary motor cortex (M1) stimulation. However, the effects of tDCS on the cerebellar cortex should be better investigated due to divergence in the study results. Are recognized that both early and late blind people have morphological changes in their whole brain, and the cerebellum is one of these structures. For example, Kosif et al. (8) showed a smaller volume of cerebellar area in congenital blindness subjects compared to sighted subjects. But systematic review studies showed positive effects of cerebellar tDCS to modulate cerebellocerebral networks (9, 10). But until the present, no study has investigated the effects of cerebellar tDCS on gait and PC in blind people.

As stated earlier for the visually impaired people, motor control is quite challenged even in simple tasks that require PC due to their blindness, and that tDCS induces cerebellar excitability changes (11), in which can modulate cerebellar-motor cortex excitability and thus, affect positively PC processing (12). The present study hypothesizes that tDCS can potentiate the effects of a proprioceptive exercises on both static and dynamic PC in blind subjects. The secondary objectives are (1) to determine possible differences in PC betweenindividuals with congenital and acquired blindness; (2) to evaluate the motor pattern of gait with and without the use of a guide stick when wearing shoes or while barefoot; and (3) to analyze whether proprioceptive exercises with or without tDCS can lead to different results with regard to PC.

## Materials and Methods

### Study Design

This study protocol is divided into three phases where phase 1 is a cross-sectional study to determine differences in postural control and gait between groups; phase 2 is a pilot study of phase three, a randomized, controlled, double-blind, clinical trial with blind individuals.

### Ethical Approval and Consent to Participate

All procedures were performed following the Guidelines and Regulating Norms of Research involving human beings, formulated by the Brazilian National Health Council—Health Ministry of Brazil, established in 2012. This study received approval from the Human Research Ethics Committee of the Nove de Julho Educational Association (No. 1.672.635/2016). The study protocol was registered in the Clinical Trial Registration (number NCT03173105). All participants and/or their person in charge must agree to take part in the study by signing an Informed Consent Form. In addition, any personal data as well as the participants' identities, based on the ethical principles of confidentiality and privacy, will be guaranteed absolute secrecy. Sham intervention procedures will always be performed in association with an active treatment, which makes its use of less impact to the patient.

### Sample and Recruitment

Preselected individuals will undergo a general assessment, with personal information, and anthropometric measurements which will be kept confidentiality under the responsibility of the researcher for the study.

**Figure 1:**
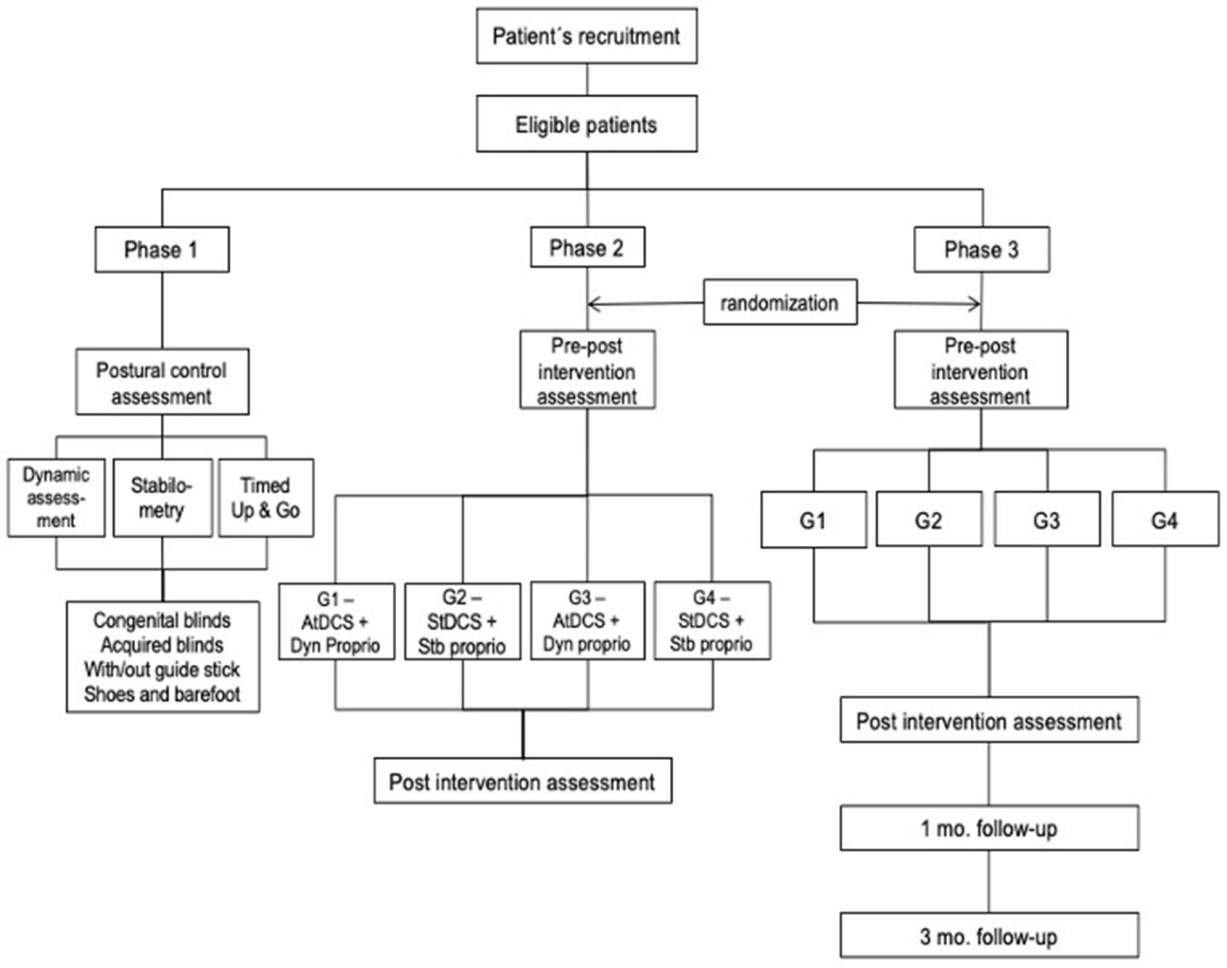
Flowchart of the study design. AtDCS, active transcranial direct current stimulation; StDCS, *sham* transcranial direct current stimulation; Dyn proprio, dynamic proprioception; Stb proprio, static proprioception.

The study will be conducted in three phases: (1) to determine differences in PC and gait between individuals with congenital and acquired blindness, and with/without a guide stick when wearing shoes and while barefoot compared to sighted people; (2) a pilot study to observe the effects of a cerebellar anodal tDCS on PC and gait during a proprioceptive exercises protocol; and (3) the exercise protocol with sample size calculation from phase 2. Figure 1 displays the flowchart of the proposed study process.

Volunteers will be recruited from the community and associations that offer assistance to individuals with visual impairment. Letters will also be sent to specialized healthcare professionals to divulge the project. Some strategies will be employed to increase participation besides decreasing the dropout rate and achieve adequate participants to reach target sample size.

**Figure 2:**
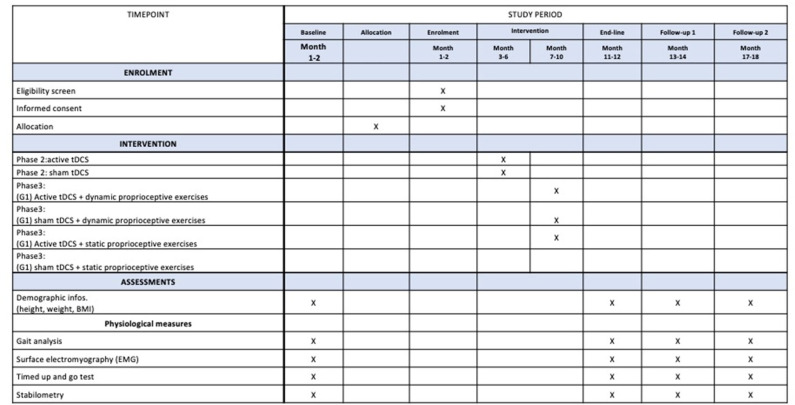
Schedule of enrollment, assessments, and interventions for participants.

The characterization of congenital and acquired blindness will be based on the classification of the degree of visual impairment proposed by the World Health Organization, the International Statistical Classification of Disease and the 10th Edition of the International Classification of Disease, in which visual acuity <20/400 or <20/200 in the better eye is classified as blindness. The time schedule for trial enrollment, interventions, assessments, and participant visits is provided in Figure 2.

### Eligibility Criteria

Inclusion criteria will be abnormalities of the optic nerve, retina disorders, glaucoma, Stargardt disease, macular degeneration, retinitis pigmentosa, congenital toxoplasmosis, congenital cataracts, congenital Leber's amaurosis, detached retina, and astrocytoma. For exclusion criteria: medical diagnosis of an injury, surgery, or clinical condition that can affect balance and gait; the use of medication affecting the central nervous system, coordination or balance; current symptoms of vertigo or dizziness. Additional exclusion criteria that should be considered for tDCS are: frequent migraines/headaches, scalp or skin conditions, metallic implants within the body, and seizures.

### Evaluation Procedures

All evaluations will be done at baseline and post-immediately therapeutic intervention, Also in 1 and 3 months as follow-ups (Figure 1).

#### Three-Dimensional Gait Analysis

Gait will be analyzed on a track measuring 90 centimeters in width and five meters in length. The SMART-D 140 system (BTS Engineering, Milan, Italy) will be used, which has eight infrared light-sensitive cameras and synchronized to a video system and computer containing the SMART-D INTEGRATED WORKSTATION® with 32 analog channels. Two force-platforms (Kistler, model 9286BA) will be used for the collection of kinematic gait data, the recording of the displacement of the center of pressure and the contact time between the foot and the surface of the force-platform. To ensure consistency in the results, at least six steps will be performed during each evaluation. The following indices will be analyzed based on the kinematic, kinetic: spatiotemporal variables (velocity, cadence, step length, stride length, stance phase, and swing face) and joint angles at specific moments of gait (pelvis inclination, hip flexion-extension, knee flexion-extension, and ankle dorsiflexion-plantar flexion) as well foot progression.

#### Surface Electromyography (sEMG) Activity

The sEMG (BTS Engineering, Milan, Italy) analysis of the right tibialis anterior, lateral gastrocnemius, rectus femoris, biceps femoris, rectus abdominal, lumbar paravertebral, sternocleidomastoids, and upper trapezius muscles will be performed, with a bioelectric signal amplifier, wireless data transmission and bipolar electrodes with a total gain of 2,000 within a frequency of 20–450 Hz. The impedance and the common rejection mode ratio of the equipment are >1,015 Ω//0.2 pF and 60/10 Hz 92 dB, respectively. All electromyographic data will be captured and digitized in 1,000 frames/second software program (BTS MYOLAB, Milan, Italy) and collected simultaneously to the kinematic and kinetic data.

#### Timed Up and Go (TUG) Test

The participants will be instructed to perform the test at a safe, self-selected pace. Blind subjects will be guided under the voice command of the researcher signaling the moment to turn around, and then to return to a seated position in the chair. Velcro strips were attached to the wall ~12–18 inches from the chair to serve as a “prompt” that the chair was nearby. The participants will be instructed to perform this task at a safe pace but were told that their time was recorded (13). The TUG test will be performed with shoes and barefoot with and without a guide stick.

#### Stabilometry

The acquisition frequency of the force-platform will be 50 Hz, captured by four piezoelectric sensors measuring 400/600 mm positioned at the extremities of each force-platform (BTS Infinity-T, Milan, Italy). The data will be recorded and interpreted using the SWAY software program (BTS 161 Engineering) integrated and synchronized to the SMART-D 140 system (BTS MYOLAB, Milan, Italy). The participants will be instructed to remain in a quiet standing position with arms alongside the body and head held in the vertical position. Three measurements (45 s each) will be performed to collect postural balance of COP (95% confidence ellipse area; velocity, and RMS in anteroposterior and mediolateral directions), and COP–COG in each condition (hard and soft surface).

### Intervention Procedure

tDCS (Trans-Cranial Technologies, USA) will be administered simultaneously during the proprioceptive exercises, ~20 min by specialized physical therapist, with two non-metallic sponge (5 × 7 cm^2^) moistened with a saline solution (15–140 mM). Anodal tDCS will be administered over the cerebellum positioned centrally located 1.5 cm below the inion, and the cathode electrode will be positioned centrally on the forehead (over Cz) (14). A current of 2 mA (0.06 mA/cm^2^) will be used during each proprioceptive exercise session. For sham tDCS, the electrodes will be positioned as described, but the stimulator will be switched on only for the first 30 s, giving the participant the initial sensation of tDCS, but without active stimulation throughout the remainder of the session.

#### Adverse Effects

The adverse effects of tDCS will be evaluated at the end of each session using a questionnaire addressing the perception of symptoms that might occur during the session, such as tingling, burning sensations, headache, pain at the electrode sites, drowsiness, and mood alterations.

#### Proprioceptive Exercises

The intervention will be divided into static and dynamic proprioceptive exercises. The static exercises will be conducted as follows: (1) standing on the toes with the feet apart; (2) standing on the toes with the feet together; (3) standing on only the right leg without support; (4) standing on only the left leg without support; and (5) standing with the heel of the right (or left) foot touching the toes of the left (or right) foot with the feet in a straight line (tandem). Exercises will be performed on an unstable surface (wobble board) on the anteroposterior (three sets) and laterolateral (three sets) axes. Each exercise will be performed in six sets of 30 s each, with a 1-min rest between sets. For dynamic proprioceptive exercises: (1) walking slowly then more quickly on a trampoline; (2) walking backward with one foot behind the other; (3) walking forward on a beam; (4) going up and down a flight of stairs; and (5) sitting on a Swiss exercise ball (65 cm) and performing laterolateral and anteroposterior movements, circling movements and bouncing. Activities 1–4 will be performed in three 1-min sets, and activity five will be performed in sets with 30 s of each movement. Throughout all exercises, a physical therapist will remain beside the participant to avoid excessive imbalance and the risk of falls.

### Sample Size Estimation

The sample size will be estimated from Phase II where participants will be randomly allocated into four groups (10 each group). Then the calculation will be considering the minimal difference between the mean of an analysis of variance results obtained from both gait speed and the displacement of the center of pressure (COP) as the primary outcome. Thus, the sample size will be estimated with a unidirectional alpha of 0.05 and an 85% statistical power. The sample determined by the calculation will be increased by 20% to compensate for possible dropouts.

### Randomization

A randomization in blocks will be applied, in which the participants will be randomly allocated to one of the four groups: (G1 = active tDCS + dynamic proprioceptive exercises; G2 = sham tDCS + dynamic proprioceptive exercises; G3 = active tDCS + static proprioceptive exercises; and G4 = sham tDCS + static proprioceptive exercises). Randomization will be done at the website www.random.org and conducting by a person blind to the objectives and protocol of this study.

### Group Allocation

An opaque and sealed envelopes will be used to conceal group information. After signing the informed consent, individuals will choose an envelope with the name of the group to which they will be assigned in phases II and III. This step will be managed by a third person who is not part of the study.

### Blinding

The physical therapist that will conduct the evaluations will be blinded to the group assignment and to the objectives of the study.

To blind the sham group the stimulator will be turned on for only 30 s and then turned off. In this way, the subjects will have the initial sensation associated with electrostimulation but will not receive any stimulation in the remaining time.

### Statistical Analysis

The data will be analyzed by Shapiro-wilk test to determine adherence to the Gaussian curve. If the parametric distribution is confirmed, the data will be expressed as mean and standard deviation, and for non-parametric variables, data will be expressed as the median and interquartile range. The effect size will be calculated based on the difference between means of the preintervention and post-intervention evaluations and will be expressed with their respective 95% confidence intervals.

An ANOVA or the Kruskal-Wallis test will be used for the analysis of the effects obtained in the phases one and two of the study. The Bonferroni correction for multiple comparisons will be employed as a *post-hoc* test. The analyses will be performed considering spatiotemporal gait variables, variables of COP parameters, and EMG signals as the dependent variables. The fixed independent variables in Phase I will be group (congenital and acquired blindness, and sighted), auxiliary resource (with and without guide stick), and shoes (with and without). In Phase II and III, the dependent variables will be the same as described in phase I, and for the fixed independent variables will be groups plus active and sham tDCS groups, evaluation time (preintervention, post-intervention, and follow-up) and group^*^evaluation time interaction. *P* < 0.05 will be considered indicative of statistical significance. The data will be analyzed using the Statistical Package for the Social Sciences (SPSS v.19.0) and Biostat (Analystsoft v. 2008.5.0.1).

## Discussion

To date, little has been written on the effects of proprioceptive exercises on improvements in PC and balance in individuals with blindness. Rogge et al. (15) showed that balance training is capable to improve balance performance in blind adults after 6 weeks at various types of balance exercises. Others have indirectly cited in studies quantifying PC in these individuals using moving platforms, with reports of improvements in balance following the sessions (1, 3). Thus, the present study proposes a rehabilitation protocol for improving PC in blind individuals involving proprioceptive exercises combined with the administration of tDCS.

According to Schmid et al. (1) improved proprioceptive and synesthetic functions as a result of a long process of neural plasticity in blind individuals do not compensate for the contribution of vision to locomotion. Evaluating motor control performance with and without sudden postural perturbations in blind individuals and those with normal vision, it was seen that PC was significantly poorer in the former group, even among those with better proprioceptive acuity (4, 16). The authors concluded that superior proprioceptive acuity does not translate to better balance control. Moreover, blind individuals showed a greater fear of falling.

The main source of information from the surrounding environment is visual stimuli, which is integrated with other sensory inputs for the selection of a balance strategy (1, 3). Alghadir et al. (17) found that the reduction in visual information in individuals with poor vision did not affect postural stability on a soft surface in comparison to a group with normal vision. Duarte and Zatsiorsky (18) reported that proprioceptive information from the sole of the foot is likely limited, and the PC system would need to employ the visual and vestibular systems more to control balance. Häkkinen et al. (19) suggested that blind individuals use proprioceptors more for balance control, whereas individuals with normal vision rely mainly on visual signals.

It seems that specific training, besides improving balance, also induces neural plasticity in brain regions associated with somatosensory and vestibular processing (3, 15). This improvement in balance performance in blind subjects could be related to specific training that stimulate the neuroplasticity of some brain structures (15, 20, 21). Taken all together, the proposal of a proprioceptive therapeutic intervention combined with tDCS is an innovative method. Clinically, tDCS is attractive due to its low cost and easy use as well as its safe, and non-invasive administration (22). The capacity to generate changes in the excitability of neural tissue that can lead to neuroplastic changes and the combined use of tDCS with other rehabilitation therapies makes this method a very interesting option when the aim is to enhance therapeutic results (8).

## Data Availability Statement

The raw data supporting the conclusions of this article will be made available by the authors, without undue reservation.

## Ethics Statement

This study protocol was reviewed and approved by the Human Research Ethics Committee of the Nove de Julho Educational Association (No. 1.672.635/2016). The study protocol was registered in the Clinical Trial Registration (number NCT03173105). Written informed consent will be provided by the participants and/or their legal guardian/next of kin.

## Author Contributions

RP and CO conceived and wrote the study. RP, CO, JL, MA, DC, LC, and MF prepared the manuscript and participated in the study design. JL, MG, and VC reviewed and approved the manuscript for final submission. All authors contributed to the article and approved the submitted version.

## Conflict of Interest

The authors declare that the research was conducted in the absence of any commercial or financial relationships that could be construed as a potential conflict of interest.
